# Anxiety and depression disorders in Portugal during the COVID-19 pandemic: Juan Rachadell

**DOI:** 10.1093/eurpub/ckac131.486

**Published:** 2022-10-25

**Authors:** A Santos, J Rachadell

**Affiliations:** 1 Public Health Unit, ACES Almada Seixal, Almada, Portugal; 2 Public Health Unit, ACES Lisboa Ocidental e Oeiras, Oeiras, Portugal; 3 EUPHA-DH; 4 Institute for Evidence Based Health, Lisbon, Portugal

## Abstract

**Background:**

Mental disorders are a major contributor to the global burden of disease. During the first year of the pandemic by COVID-19, increases of 25% in the prevalence of anxiety and depression were reported globally. In Portugal, studies conducted during lockdown showed an increase in the prevalence of these disorders. However, previous studies have shown that negative life events, such as exposure to disasters or grief, later lead to resilience or recovery. It is therefore necessary to study the evolution of these disorders in order to adapt mental health measures.

**Methods:**

The number of patients registered with “P76 - Depressive Disorder” and “P74 - Anxiety Disorder/anxiety state”, according to ICPC-2 criteria, and the total number of patients registered in the Portuguese Health Centers for the months of January 2019 to 2022 were obtained from the Portuguese NHS Information and Monitoring System (SIM@SNS). We calculated the percentage of patients with each of the disorders, individually and combined. Data by health regions were also obtained in order to compare the evolution within each region (North, Center, Lisbon and Tagus Valley, Alentejo and Algarve).

**Results:**

Between January 2019 and 2022, the proportion of patients with anxiety disorder increased linearly from 8% to 9%. Similarly, the proportion of patients with depressive disorder increased from 11% to 12%. When considered together, anxiety and depression disorders affected 21% of users in mainland Portugal at the beginning of 2022. The Alentejo and Center regions have the highest prevalence of anxiety and depression (24.9% and 24%, respectively) and the Algarve region has the lowest (16.74%). The increases were consistent across health regions, with the largest increase in the North region (2.6%) and the smallest in the Central region (1.7%).

**Conclusions:**

Anxiety and depression disorders increased in mainland Portugal during the years of the COVID-19 pandemic.

**Key messages:**

• Mental health has been an important factor in public health since before the pandemic.

• Monitoring depression and anxiety levels in the general public can guide priorities after the pandemic.

